# Pancreatic Cystic Lesions: From Basic Knowledge to Recent Guidelines

**DOI:** 10.3390/jcm15020585

**Published:** 2026-01-11

**Authors:** Ginevra Danti, Ludovica Scalzone, Lavinia Mattolini, Matilde Anichini, Francesca Treballi, Linda Calistri, Diletta Cozzi, Vittorio Miele

**Affiliations:** 1 Department of Radiology, Careggi University Hospital, Largo Brambilla 3, 50134 Florence, Italy; ginevra.danti@gmail.com (G.D.); scalzoneludovica@gmail.com (L.S.); mattolini.lavinia@gmail.com (L.M.); matyanichini@gmail.com (M.A.); francescatreballi@gmail.com (F.T.); linda.calistri@unifi.it (L.C.); dilettacozzi@gmail.com (D.C.); 2 Department of Experimental and Clinical Biomedical Sciences “Mario Serio”, University of Florence, 50121 Florence, Italy

**Keywords:** pancreatic cystic lesions, PCLs, radiomics

## Abstract

Pancreatic cystic lesions (PCLs) are increasingly detected due to widespread use of cross-sectional imaging. They encompass a heterogeneous group of lesions, ranging from benign pseudocysts and serous cystic neoplasms (SCNs) to premalignant mucinous cystic neoplasms (MCNs) and intraductal papillary mucinous neoplasms (IPMNs), as well as rare malignant entities such as solid pseudopapillary epithelial neoplasm (SPENs) and cystic pancreatic neuroendocrine tumors (cystic PanNETs). Management of PCLs depends on their malignant potential; therefore, an accurate classification is essential for optimizing treatment. This narrative review summarizes current knowledge on the epidemiology, imaging characteristics, diagnosis, and management of PCLs, highlighting the role of CT, MRI, MRCP, and endoscopic ultrasound. Recent advances in radiomics for lesion characterization and risk stratification, particularly in IPMNs, are discussed.

## 1. Introduction

Pancreatic cystic lesions (PCLs) are defined as well-circumscribed, fluid-filled lesions of the pancreas. Their reported incidence has been increasing in recent years, primarily due to the widespread use of cross-sectional imaging modalities such as CT and MRI, through which they are frequently identified incidentally. Despite their relatively low probability of malignant progression, the potential for neoplastic transformation often necessitates additional diagnostic work-up [1].

According to a 2024 systematic review and meta-analysis on the global prevalence of pancreatic cystic lesions (including both incidental findings and clinically relevant cysts), these lesions are present in 13% to 18% of the population [2], with incidence rising progressively across older age groups [3,4].

Following the WHO criteria, PCLs can be categorized into four subgroups based on the presence or absence of an epithelial lining and their benign or malignant nature [5].

Pseudocystic lesions, which lack an epithelial wall and are benign, account for approximately 75% of all PCLs. The remaining 25% consists of neoplasms (10%), congestive/retention cyst (10%), and congenital cysts (including polycystic disease, cystic fibrosis, and cystic pancreatic lymphangioma, also comprising about 10% [6,7,8].

Cystic neoplasms may cause nonspecific gastrointestinal symptoms, such as abdominal pain, nausea, and dyspepsia; therefore, their detection is rarely based on clinical evidence and it is more commonly incidental [6].

Given the heterogeneity of these lesions, establishing an accurate differential diagnosis is essential, as certain subtypes—particularly intraductal papillary mucinous neoplasms and mucinous cystic neoplasms—are associated with a certain risk of malignant transformation. Consequently, appropriate diagnostic assessment and management are critical [7].

## 2. Imaging Techniques

Transabdominal ultrasound (TUS) is frequently the first imaging approach in the evaluation of pancreatic pathology; however, US assessment of the pancreas is frequently limited by overlying bowel gas, particularly when attempting to detect or characterize lesions located in the pancreatic body and tail.

Multidetector computed tomography (MDCT) and magnetic resonance (MRI) with cholangiopancreatography provide more reliable visualization for this purpose.

MDCT can detect main pancreatic duct (MPD) dilatation, cysts morphology, wall thickness and intracystic nodules.

Nevertheless, MRI is considered the gold standard for both diagnosis and follow-up of PCLs, as it allows clinicians to identify mural nodules, septa and dilation of the main pancreatic duct, in addition to characterizing the signal properties of intralesional components.

When combined with cholangiopancreatography, MRI also facilitates assessment of potential connection between cystic lesions and the pancreatic ductal system [9].

## 3. Pseudocyst

Pseudocysts are fluid collections rich in amylase, defined as “pseudo” because they lack an epithelial lining: the fluid is in fact surrounded by a non-epithelialized wall made up of fibrosis and granulation tissue. Their incidence is estimated at 0.5–1 per 100.000 adults [10]. Although pseudocysts may arise following trauma or surgery, they most commonly develop as a sequela of both acute and chronic pancreatitis [11].

In these cases, the inflammation leads to the release of proteolytic enzymes into the peritoneal cavity; consequently, pseudocysts typically develop 4–6 weeks after the initial insult and are most commonly located in the pancreatic tail [12].

Patients often present with non-specific symptoms such as abdominal pain, nausea, and vomiting, although fever may occur in cases complicated by infection.

Diagnosis is typically established with contrast-enhanced CT, which shows a round fluid collection with either a thin wall or a uniformly thickened wall [13] CT al-so allows differentiation between pseudocyst and walled-off-necrosis, although distinguishing pseudocysts from cystic neoplasms can remain challenging [10].

On MRI, T2w sequences may reveal non-enhancing internal debris, a highly specific imaging feature for the diagnosis of pseudocysts [14].

## 4. Serous Cystic Lesions or Neoplasm (SCN)

SCNs account for approximately one-third of primary pancreatic cystic neoplasms (PCNs). They are almost always benign and are predominantly located in the pancreatic body and tail.

SCNs occur more frequently in women between 50 and 70 years of age [15,16]. Although the exact aetiology remains uncertain, an association with certain genetic disorders—most notably Von Hippel–Lindau (VHL) syndrome—has been described [17].

**Figure 1:**
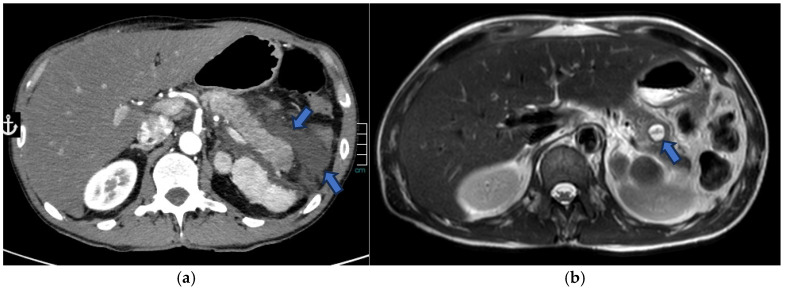
(**a**) Pseudocyst in a 49-year-old man: axial contrast enhanced CT scans show a free fluid collections surrounding the body and tail of the pancreas (arrow), which appears diffusely enlarged with loss of the normal lobulated contour. (**b**) MRI was performed four weeks later, demonstrating a well-defined, round cystic lesion located in the tail of the pancreas (arrow), which shows homogeneous high signal intensity on T2w images.

**Figure 2:**
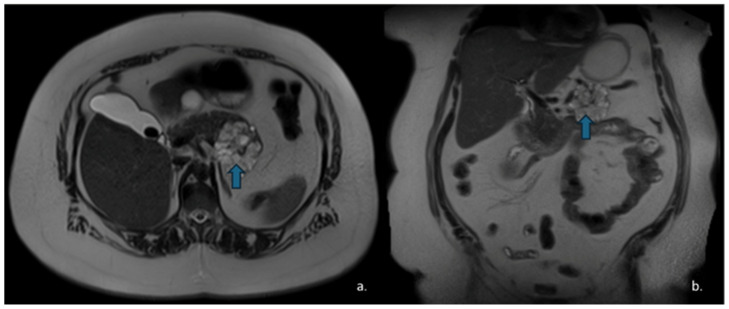
Axial (**a**) and coronal (**b**) T2-weighted HASTE sequences show a hyperintense lesion (arrow) located at the head–body passage of the pancreas. The lesion is characterized by thin, hypointense internal septa. Endoscopic ultrasound revealed a microcystic serous cystadenoma.

**Figure 3:**
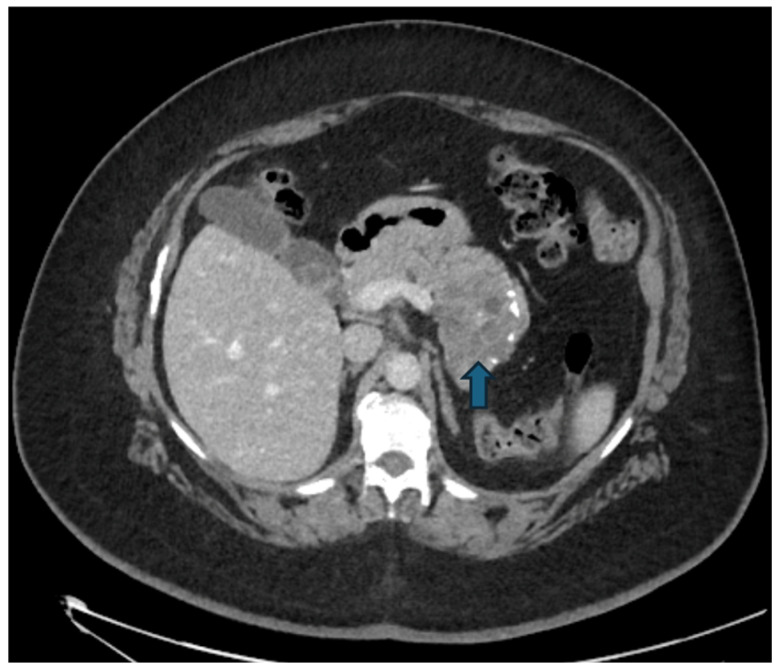
CT scan (portal venous phase) of a 67-year-old woman. A hypodense lesion is observed in the pancreatic body (arrow), with numerous thin, partially calcified internal septa, producing a characteristic “honeycomb” appearance, consistent with a microcystic serous cystadenoma.

SCNs exhibit quite heterogeneous appearance: they may present as microcystic or “honeycomb” lesions, the most common pattern (approximately 45%) (Figure 1 and Figure 2); macrocystic or oligocystic forms (32%) (Figure 3); mixed microcystic–macrocystic variants (18%); or, less commonly, solid-appearing forms (5%) [16].

**Figure 4:**
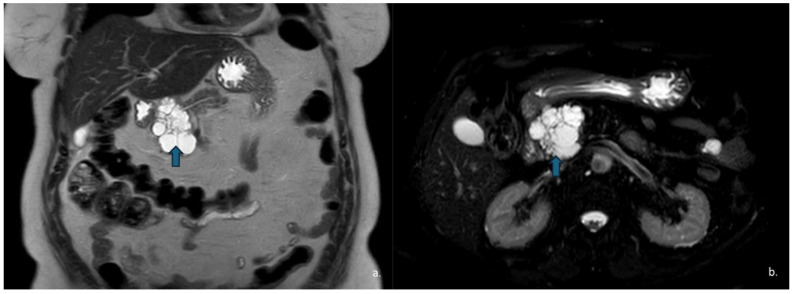
MRI of a 51-year-old woman. Coronal T2-w HASTE (**a**) and axial T2-w SPAIR (**b**) sequences show a hyperintense lesion (arrows) in the pancreatic head with a “cluster” morphology. Endoscopic ultrasound confirmed the diagnosis of a macrocystic serous cystadenoma.

On unenhanced CT scans, SCNs typically appear as lobulated and quite hypodense lesions, depending on the amount of fluid inside the cysts. Calcifications are pathognomonic, and in the microcystic variant (defined by cysts measuring <2 cm), a hyperdense central star-like scar is frequently observed [18,19] (Figure 4).

Hypervascular enhancement may be observed, particularly in microcystic SCNs, which can complicate the differential diagnosis with cystic neuroendocrine tumours (NETs) [20].

Meanwhile, macrocystic SCNs, which are made up of a little number of cysts, may be difficult to distinguish from pseudocysts or mucinous cystic neoplasms [21].

MRI is pivotal for further characterization of these lesions. On T1-weighted (T1w) images, the cystic fluid typically appears hypointense, whereas it is hyperintense on T2w sequences.

Small intralesional hemorrhages may result in hyperintense signal on T1w images. The central fibrous scar is hypointense on both T1w and T2w images. SCNs generally do not communicate with the pancreatic ducts, a feature best evaluated with MRCP [1,22].

Endoscopic ultrasound (EUS) with fine-needle aspiration can provide definitive diagnosis through the analysis of carcinoembryonic antigen (CEA) and amylase levels. Since these lesions are non-mucinous and lack ductal communication, both amylases and CEA levels are typically low (CEA < 192 ng/mL) [23].

## 5. Intraductal Papillary Mucinous Neoplasm (IPMN)

Intraductal papillary mucinous neoplasms (IPMNs) are cystic neoplasms of the pancreas that arise from epithelial cells lining the main pancreatic duct, the branch ducts, or both. IPMNs are grossly visible intraductal epithelial tumors composed of mucin-producing columnar cells exhibiting papillary proliferation and cystic dilation. Tumour cells grow up producing mucin, leading to dilation of the main pancreatic duct alone (MD-IPMN), the branch ducts alone (BD-IPMN), or both (mixed-type IPMN) [24].

IPMNs show a slight male predominance and most commonly occur in older individuals (median age, 65 years) [25,26].

Approximately 80% of incidentally discovered pancreatic cysts are considered to be branch-duct IPMNs (BD-IPMNs) [27,28].

**Figure 5:**
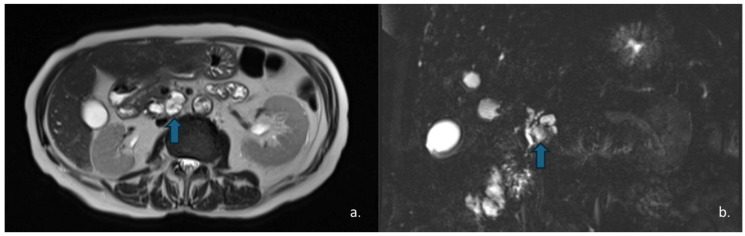
MRI T2-w HASTE (**a**) and MR cholangiographic (**b**) sequences show a hyperintense lesion at the level of the pancreatic uncinate process (arrow). The cholangiographic sequence demonstrates communication with the main pancreatic duct (duct of Wirsung), consistent with a type II intraductal papillary mucinous neoplasm (IPMN).

They are considered benign lesions with malignant potential, which increases with age. IPMNs diagnosed in older patients are more likely to progress to high-risk stigmata, although age itself does not appear to influence cancer-related mortality [29] (Figure 5).

Patients with IPMN—particularly those with MD-IPMN—may present with recurrent episodes of pancreatitis or with a clinical picture resembling “idiopathic” chronic pancreatitis. Episodes of acute or chronic pancreatitis are caused by mucus accumulation or papillary projections within the pancreatic duct, causing an obstruction of the ductal system. BD-IPMNs are most often asymptomatic, especially when their diameter is <3 cm. Clinical manifestations typical of pancreatic adenocarcinoma (such as weight loss or jaundice) may occur in cases of malignant transformation [30].

IPMNs communicate with the main pancreatic duct, a feature that can be demonstrated by MRCP [31] and is useful for distinguishing them from other pancreatic cystic lesions. MD-IPMNs are characterized by isolated involvement of the main pancreatic duct with ductal dilation > 5 mm; this dilation may be diffuse or segmental and most commonly involves the pancreatic body and tail [32].

BD-IPMN presents as a cystic dilation > 5 mm arising from a side branch of the main pancreatic duct and is most commonly located in the head or uncinate process of the pancreas [30].

In some cases, it may be multicentric, involving multiple non-contiguous side branches of the main pancreatic duct [33]. Mixed-type IPMN involves both the main duct and its side branches and carries a malignancy risk comparable to that of MD-IPMN.

CEA and amylase levels may be useful but do not allow reliable differentiation between mucinous cystic neoplasms (MCNs) and IPMNs. Elevated CEA is a marker that distinguishes mucinous from non-mucinous cysts, but it does not discriminate between benign and malignant lesions. A cut-off value of ≥192–200 ng/mL has an accuracy of approximately 80% for identifying a mucinous cyst, with high specificity but low sensitivity [34,35]. Recent studies have shown that molecular analysis for GNAS mutations can differentiate BD-IPMNs from MCNs, as GNAS mutations are typically present in IPMNs but absent in MCNs [36]. Serum CA19-9 is an independent predictor of malignancy in IPMNs; values > 37 U/L are associated with an increased risk of invasive carcinoma [37,38,39].

### International Evidence-Based Kyoto Guidelines: Diagnosis and Management of IPMN

The current algorithm used for the evaluation and follow-up of IPMNs is based on the 2024 Kyoto Guidelines [40], which revised the 2017 Fukuoka guidelines [41].

The aim of the group who developed these new guidelines was to conduct a systematic review for each of the five main topics underlying the diagnostic process: revision of high-risk stigmata (HRS) and worrisome features (WFs), surveillance for non-resected IPMN, surveillance after surgical resection of IPMN, revision of pathological aspects, and investigation of molecular markers in cyst fluid (Table 1).

Regarding the WFs, two main updates were introduced, regarding the cyst growth rate and the new onset or recent exacerbation of diabetes mellitus (DM).

It has been in fact proven that the prevalence of DM is high among patients with IPMNs, and that newly diagnosed DM is frequently associated with a higher risk of aggressive disease, although the aetiology of this association remains unclear [42].

As to cystic growth rate, several studies have shown that it can be used as a predictive factor for progression to malignancy; a growth rate of ≥2.5 mm/year has been most consistently reported—replacing the previous threshold of 5 mm/2 years [43,44,45,46].

Our duty therefore is to identify the imaging features most strongly associated with the risk of developing high-grade dysplasia or invasive carcinoma.

**Figure 6:**
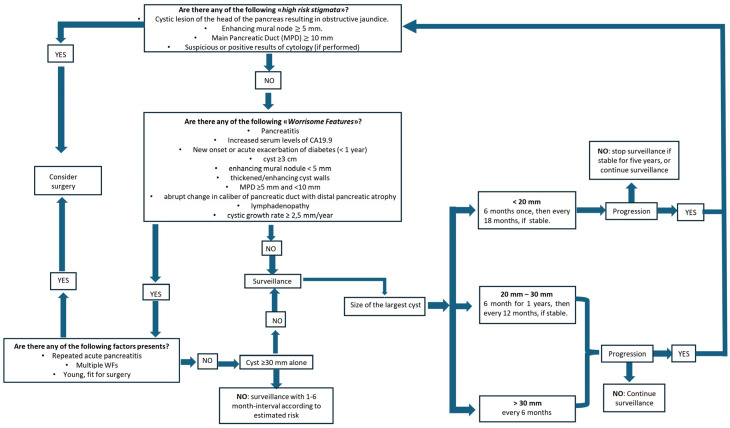
Algorithm for management of IPMN.

Figure 6 shows a clear management of IPMN answering some questions as described below:

Are any “high-risk stigmata” of malignancy present?
  ○ If the answer is yes, the management direction aims towards surgery.
  ○ If the answer is no, as often happens, we focus on the second question:
Are any “worrisome features” present?
  ○ If there aren’t any worrisome features, surveillance is required and it will depend on the size of the cyst.
  ○ If some worrisome features are present, we focus of the third question:
Are there any of the following factors present: repeated acute pancreatitis, multiple WFs, patient young and fit for surgery?
  ○ If the answer is yes, consider surgery.
  ○ If the answer is no, surveillance is required.

It is, however, important to note that the role of cyst size as a standalone criterion remains controversial. Some studies have downplayed the significance of this parameter, observing that in several surgical series of resected IPMNs, a cyst size ≥ 3 cm showed a positive predictive value (PPV) for malignancy of only 27–33% [47].

Other studies have validated the safety of observation of BD-IPMN measuring <4 cm in the absence of other risk factors [48,49]. Cyst size alone should be considered an appropriate indication for surgery, while if multiple risk factors are present, the sensitivity of size to detect malignancy increases [50].

In his meta-analysis, Marchegiani demonstrated that the presence of mural nodules (MNs) was the strongest independent predictor of invasive cancer and high-grade dysplasia (HGD) for all type of IPMNs (with the exception of HGD in BD-IPMNs). Also, the mural nodule size could be a parameter used to predict the malignancy of IP-MNs [51].

Overall, the primary limitation of the Kyoto guidelines is that many of the supporting studies lack a high and robust level of recommendation. Consequently, although these guidelines synthesize the available evidence, their practical application remains a subject of debate, particularly regarding the surveillance of non-resected BD-IPMNs [52].

Ultimately, the most significant achievement of the Kyoto guidelines has been to highlight the necessity of a management algorithm tailored specifically to the individual patient [53].

## 6. Mucinous Cystic Neoplasms (MCN)

Mucinous cystic neoplasms (MCNs) of the pancreas predominantly affect women in their 40s and 50s [1]. They are typically located in the body and tail of the pancreas and tend to be centrally positioned within the gland, unlike intraductal papillary mucinous neoplasms (IPMNs), which are peripherally located—features that aid radiologists in the differential diagnosis. MCNs contain a dense ovarian-like stroma. These lesions are considered pre-malignant, as they can progress to mucinous cystadenocarcinoma. They may reach considerable size but do not cause dilation of the main pancreatic duct [54].

**Figure 7:**
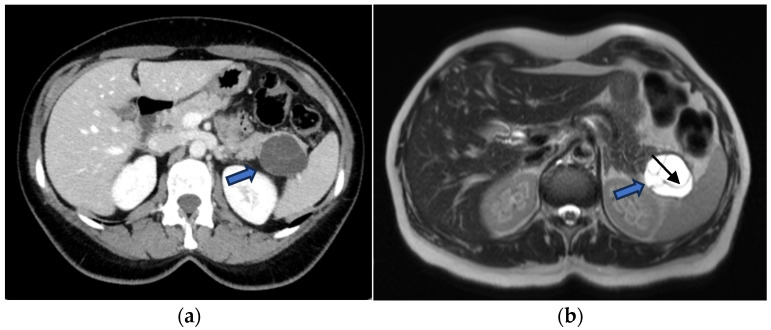
(**a**) Axial CECT scan of a 50-year-old-woman shows a large (40 mm), multiloculated lesion located in the pancreatic tail (blue arrow); (**b**) MRI demonstrates a well-circumscribed, multilocular cystic lesion with thick internal septa (black arrow), showing hyperintense signal on T2-w images.

MCNs typically present as multiloculated macrocystic lesions due to the presence of thick internal septa. Peripheral calcifications (found in approximately 25% of cases) are considered pathognomonic and enable a definitive diagnosis (Figure 7).

**Figure 8:**
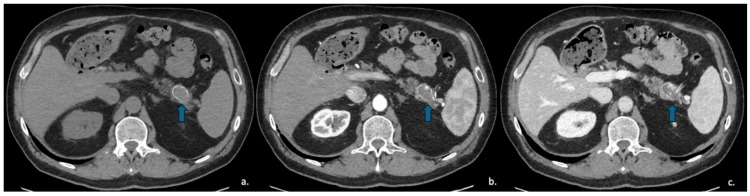
CECT of a 49-year-old woman shows a cyst-like lesion with a calcified wall (arrow) in the pancreatic tail (**a**). In the arterial (**b**) and venous (**c**) contrastographic phases, mall enhancing mural nodules are observed.

The primary differential diagnosis for MCNs is IPMN, and distinguishing between the two is essential, as type II IPMNs generally require imaging surveillance, whereas most MCNs require surgical resection [54].

Typically, the identification of a cystic lesion centrally located within the gland, without dilation of the main pancreatic duct, in a middle-aged woman should raise suspicion for MCN.

EUS is particularly useful in this setting, as it enables direct visualization of a cystic lesion that lacks communication with the pancreatic duct.

Fine-needle aspiration (FNA) typically reveals mucinous epithelium, while analysis of the intralesional fluid demonstrates elevated CEA levels (>192 ng/mL) and low amylase levels [55]: this pattern definitively confirms that the lesion is mucinous (high CEA) and lacks communication with the pancreatic duct (low amylase) (Figure 8).

## 7. Solid Pseudopapillary Epithelial Neoplasm (SPEN)

Solid pseudopapillary epithelial neoplasm (SPEN) is a rare pancreatic tumor, accounting for <3% of all pancreatic neoplasms, and is characterized by a low malignant potential (<10% risk of metastasis or recurrence).

It occurs almost exclusively in young patients (<35 years) and predominantly in women (>90%). The etiology remains unknown, although some cases have been reported in association with familial adenomatous polyposis (FAP) [56]. SPENs are generally asymptomatic and incidentally found on imaging; however, some patients may present with mild symptoms such as abdominal pain, nausea, vomiting or asthenia [57].

Serum levels of tumor markers such as CEA, CA19-9, and α-FP (47) are typically within the normal range and therefore are not useful for diagnosis [58].

SPENs may arise in any part of the pancreas, although they are more frequently located in the tail. They can also occur in extra-pancreatic sites, such as omentum, mesentery, retroperitoneum, ovary, stomach or duodenum. On CT, SPENs appear as well-demarcated, large heterogeneous masses with mixed solid and cystic areas and hemorrhagic degeneration: the enhancing solid portions are typically peripheral, while the cystic areas tend to be centrally located.

**Figure 9:**
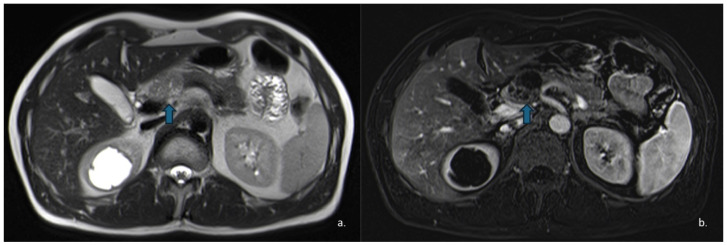
MRI of a 37-year-old woman shows a lesion in the pancreatic head (arrow) with intermediate signal intensity on T2-w HASTE sequences (**a**) and mild enhancement of the solid component on dynamic arterial-phase sequence (**b**). Endoscopic ultrasound suggested the diagnosis of a solid pseudopapillary epithelial neoplasm (SPEN).

Peripheral or central stippled calcifications may also be present [59]. On MRI, SPENs usually present as well-defined masses with heterogeneous signal intensity on both T1w and T2w sequences, reflecting their combined solid and cystic structure. High T1 signal intensity may be observed due to areas of hemorrhagic necrosis or internal debris. T2 signal intensity is variable, related to the presence of different hemoglobin degradation products. The solid components typically demonstrate iso- to low signal intensity on T1w images and slightly high signal intensity on T2w images [60,61] (Figure 9).

## 8. Cystic Pancreatic Neuroendocrine Tumour (PanNET)

**Figure 10:**
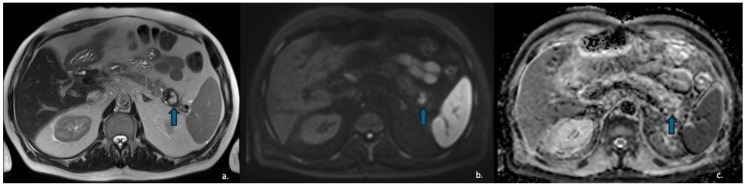
The same patient as in Figure 7 underwent Contrast-Enhanced MRI, which showed a lesion (arrow) with inhomogeneous signal in the T2w—HASTE sequences (**a**) and areas of restricted diffusion in DWI and ADC maps corresponding to the mural nodules. (**b**,**c**) The patient subsequently underwent surgical resection, and histopathology confirmed a degenerated mucinous cystadenoma.

Pancreatic neuroendocrine tumours (PanNETs) are relatively rare neoplasms, with an annual prevalence of approximately 0.8 per million individuals. They typically present as solid lesions, although a cystic appearance may occasionally occur. Cystic PanNETs are believed to arise from degenerative processes, such as necrosis or ischemia within an initially solid PanNET [62] (Figure 10). When PanNETs exhibit a cystic morphology, they are usually non-functional, relatively small [63] and most commonly located in the body and tail of the pancreas [64]. Overall, cystic PanNETs demonstrate a lower malignant potential compared with their solid counterparts; in fact, a study by Khalil et al. reported no cases of metastasis or recurrence [65].

**Figure 11:**
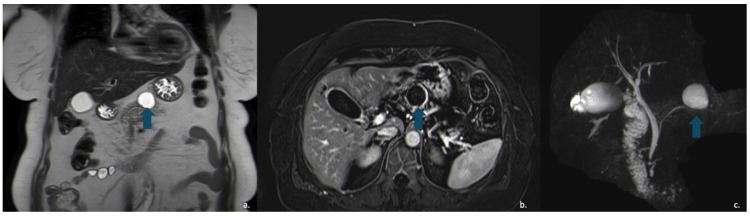
MRI of a 57-year-old man with neuroendocrine neoplasm (NEN) with cystic degeneration involving the pancreatic body (arrow). The lesion appears hyperintense on T2-w-HASTE sequences (**a**) and demonstrates peripheral rim-enhancement (**b**). MR cholangiography shows no communication with the main pancreatic duct (**c**).

On CECT, cystic PanNETs typically appear as mixed cystic and solid lesions with rim or peripheral enhancement in both the arterial and portal venous phases, reflecting their rich vascular supply—although the arterial-phase enhancement is generally more pronounced. MRI is valuable for evaluating communication with the pancreatic ducts, which is typically absent in PanNETs [66,67] (Figure 11).

Occasionally, certain PanNETs present with ill-defined margins, heterogeneous hypovascular enhancement, and main pancreatic duct dilatation, closely resembling pancreatic adenocarcinoma and complicating differential diagnosis. Nevertheless, this diagnostic difficulty can be overcome through the use of Dual-Energy CT, which facilitates tissue characterization and lesion differentiation [68].

## 9. Radiomics in Cystic Lesions of the Pancreas

Radiomics is the study of quantitative features extracted from radiologic imaging, which can improve diagnostic accuracy across a wide range of conditions, including not only oncologic diseases but also emergency settings [69,70].

In recent years, Texture Analysis (TA), a branch of radiomics, has enabled quantitative assessment of tumor heterogeneity by evaluating the distribution and spatial relationships of pixel or voxel grey levels within an image [69,71].

Various TA methods can be applied, including statistical-, model- and transformed-based methods; among these, statistical-based techniques are the most widely used, due to the availability of commercial and in-house software tools [72].

Radiomics features are mathematically calculated using first-order, second-order, or higher-order statistical tools. In particular, First-order Texture Features describe Histogram-based characteristic (e.g., Kurtosis, Skewness, Standard Deviation). Shape features—only for 3D analysis—(e.g., volume, compactness, sphericity). Second order texture features (e.g., Grey-Level Zone Length Matrix—GLZLM, Grey-Level Run Length Matrix—GLRLM, Neighborhood Grey-Level Difference Matrix—NGLDM, Grey-Level Co-occurrence Matrix—GLCM) [69,70,73,74,75].

Several studies have investigated the role of radiomics in the characterization and differentiation of pancreatic cystic lesions, with particular emphasis on risk stratification in patients with IPMNs.

We conducted a targeted review of PubMed literature from 2017 to present, focusing on the evolution of radiomics. Studies were selected based on database consistency, aiming to document the field’s transition from foundational research to possible clinical implementation.

Dmitriev et al. developed an algorithm capable of distinguishing the most common types of pancreatic cystic lesions, including IPMNs, MCNs, SCNs, and SPENs.

The model incorporated patients’ demographic data along with intensity and shape features derived from texture analysis; segmentation of the cystic lesions was performed using a semi-automatic graph-based technique, and feature selection was carried out using random forest and convolutional neural network classifiers. The study demonstrated an overall accuracy of approximately 84% in the differential diagnosis of various pancreatic cystic lesions [76].

Wei et al., in a retrospective study, evaluated 260 patients who underwent pancreatic resection for a PCL between 2007 and 2017. A total of 409 features were assessed to differentiate SCNs from other PCLs. The study found that 17 intensity- and texture-based features (e.g., Wavelet NGTDM busyness, Wavelet intensity T-Median) combined with 5 guideline-based characteristics (e.g., gender, tumor location) produced the most statistically significant model for identifying SCNs [75].

Furthermore, a 2022 retrospective study by Liao et al. included 193 patients with PCLs: 99 diagnosed with SCN, 55 with MCN, and 39 with IPMN. The aim of the study was to develop classification prediction models for these lesions using deep learning and radiomics methods. Radiological features were independently analyzed by two trained radiologists and compared with surgical outcomes. The study reported area under the curve (AUC) values of 0.916 for distinguishing SCNs from non-SCNs, and an AUC value of 0.973 for differentiating MCNs from IPMNs [77].

Yuan et al., in a 2025 multicenter study, developed and validated an artificial intelligence-powered CT model, which significantly improved radiologists’ diagnostic accuracy while reducing reporting time [78].

Regarding IPMN risk stratification, Flammia et al., in a 2023 retrospective study, used an MRI-based radiomic model to identify features associated with high-risk BD-IPMNs and WF. The study analyzed data from 50 patients who underwent contrast-enhanced MRI, with a median follow-up of 36 months. Early identification of potentially malignant IPMNs is crucial to determine the optimal timing for surgical intervention [79].

Although radiomics has emerged as a valuable tool in various fields, its application in pancreatic imaging still faces significant challenges. While radiomics can effectively differentiate between mucinous and non-mucinous pancreatic cystic lesions (PCLs) and assess cystic malignant potential, several limitations must be acknowledged [80].

First, technical variability significantly impacts reproducibility: factors such as CT acquisition protocols, reconstruction methods, slice thickness, and the use of contrast agents can all alter the manual segmentation [81].

Furthermore, there is a notable lack of external validation. Many current studies suffer from low methodological quality, relying solely on internal validation. This issue, combined with a lack of cost-effectiveness analyses and the underreporting of negative results, creates a publication bias that currently prevents radiomics from being integrated into clinical practice [80,82]. Future research addressing these critical gaps is essential to validate the clinical utility of this diagnostic tool.

**Table 2:** Characteristics of different PCLs.

	Pseudocyst	Serous Cystic Neoplasms (SNC)	MD-IPMN	BD-IPMN	Mixed-IPMN	Mucinous Cystic Neoplasms (MCN)	Solid Pseudopapillary Epithelial Neoplasm (SPEN)	Cystic Pancreatic Neuroendocrine Tumor (PanNET)
Gender, Median age	M >F Variable	F >> M 50–60 years	-	M > F 65 years	-	F > M 40–50 years	F >> M <35 years	M > F 40–60 years
Location	Any	Body and tail	Body and tail	Head and uncinate process	Any	Body and tail, centrally located	Any (more frequently in the tail)	Body and tail
Morphological characteristics	Unilocular with non-epithelialized wall	Microcystic (with «honey combing» aspect), macrocystic or mixed	Diffuse or segmental dilatation of the MPD (>5 mm)	Macrocystic, «grape-like» or «cyst by cyst» forms	Intermediate features between MD-IPMN and BD-IPMN	Multiloculated macrocystic appearance with thick internal septa	Heterogeneous masses with variably solid and cystic areas and hemorrhagic degeneration	Unilocular cystic lesions with solid component
Mural nodule/solid component	Absent	Solid forms recur in 5% of cases	May be present; they are a criterion for malignancy (enhancing nodule ≥ 5 mm: high risk stigmata)			May be present (can concern malignancy)	Irregular/thick solid component may be present.	May be present
Calcification	Rare	Pathognomonic: in microcystic variant, “central scarring” is typical	Rare			May be present (25%). Peripheral calcifications are pathognomonic	Peripheral or central stippled calcifications may be present.	Rare
Content	Fluid/hemorrhage	Serous		Mucinous		Dense ovarian stroma	Fluid/hemorrhage/necrosis	Fluid/hemorrhage/necrosis
Size	Variable	Variable: can increase over the time	5–9 mm: worrisome feature ≥10 mm: high risk stigmata	>3 cm: worrisome feature	Intermediate features between MD-IPMN and BD-IPMN	Often large. If >4 cm: surgical resection	Variable	Variable: often stable over the time
Connection with MPD	Absent	Absent		Present		Absent	Absent	Absent
CEA, amylase, Ca19.9 levels	High levels of amylase	Low levels of CEA (<192 ng/mL) Low levels of A amylase	high levels of CEA and/or CA19.9 can concern malignancy High levels of amylase/lipase can be found in pancreatitis			High levels of CEA (>192 ng/mL) Low levels of amylase	Low levels of CEA. Low levels of Ca19.9	Low levels of CEA. Low levels of Ca19.9
Symptoms	Sometimes nausea or abdominal pain	Generally asymptomatic. Growth can result in pancreatitis	Generally asymptomatic if <3 cm in size; they can also result in episodes of acute or chronic pancreatitis			Nausea or abdominal pain can be present	Generally asymptomatic	Generally asymptomatic
MRI signal	Non–enhancing debris Variable signal on T2w sequences	Hypervascular enhancement Hyperintense on T2w sequences Hypointense central scar on T1w and T2w sequences	Hyperintense on T2w sequences Enhancing mural nodule(s) concern malignancy			Hyper- or Isointense content on T2w sequences Sometimes enhancing mural nodule(s) can be present	Heterogeneous signal intensity on T1w and T2w sequences due to solid and cystic component or to debris	Rim peripheral enhancement. Heterogeneous signal intensity on T2w sequences due to its cystic and/or solid component

## 10. Management of Pancreatic Cystic Lesions: Surveillance or Surgery?

Some pancreatic cystic lesions possess malignant potential, such as mucinous cystadenomas and IPMNs, whereas serous cystic lesions and pseudocysts are benign; consequently, early diagnosis is crucial, given the high mortality associated with pancreatic cancer. Notably, several management options are now available, ranging from active surveillance to surgery [83], which may be complemented by chemotherapy or, in locally advanced pancreatic cancer, ablative techniques (such as radiofrequency ablation, microwave ablation and electroporation combined with low doses of chemo-therapeutic drugs) to improve disease-free survival [84,85]. Active surveillance typically relies on MRI and MRCP [86].

Despite the existence of biomarkers such as CEA and CA 19-9, precise classification of pancreatic cysts remains a challenge due to the absence of a ‘gold standard’ biomarker. Given that existing markers are not used routinely, clinicians should rely on a multimodal approach—combining high-resolution imaging and endoscopic techniques with CA 19-9—to improve diagnostic accuracy [87,88].

Most pseudocysts resolve with conservative management, as they often resolve spontaneously [10]; however, intervention is required in the presence of symptoms (e.g., abdominal obstruction) or complications (e.g., infection, rupture, or biliary obstruction). Among the invasive approaches (percutaneous, surgical, or endoscopic drainage), the endoscopic method is preferred due to its high success rate and lower invasiveness compared with surgery. This approach involves creating a communication between the pseudocyst and the lumen of the stomach or duodenum, allowing drainage of the cystic fluid [12].

Serous cystic neoplasms (SCNs) are most likely to be benign, with a risk of malignant transformation of less than 1%; therefore, surgical resection is generally reserved for larger cysts (>4 cm) that produce mass effect symptoms on the stomach or duodenum [21] or for cysts with rapid growth rate, that may lead to pancreatitis due to the obstruction of the main pancreatic duct [21].

Asymptomatic patients should undergo annual imaging follow-up with MRI; further imaging-based follow-up is only required if symptoms develop [49].

As previously described, the management of IPMNs is guided by the Kyoto guidelines, which consider the presence of worrisome features or high-risk stigmata to determine whether imaging follow-up with MRCP or surgical resection is appropriate [40]. All patients with MD-IPMN should be considered for surgical intervention.

For mucinous cystic neoplasms (MCNs) lacking malignant features, surgical resection is typically recommended for lesions ≥ 40 mm; however, surgery is also indicated for symptomatic lesions of any size or for those demonstrating risk factors, particularly the presence of mural nodules [16,89].

Lesions smaller than 40 mm that are asymptomatic and lack specific risk factors undergo imaging follow-up, typically with MRI, EUS, or both, every six months during the first year, and annually thereafter if no changes are observed. Lifelong follow-up for these lesions is advised for patients who remain suitable candidates for surgery.

When surgery is required, anatomical resection is preferred, performed either as a left-sided pancreatectomy or a Whipple procedure. These patients are often relatively young and have preserved pancreatic parenchyma, making them excellent candidates for minimally invasive approaches [21,54]; however, it should be noted that open surgery remains the standard of care in many clinical settings, as minimally invasive techniques for pancreatic resections are not yet universally widespread and require significant expertise.

Solid pseudopapillary neoplasms (SPENs) are malignant lesions but are associated with excellent long-term survival, reaching 96% at 10 years, even in the presence of metastases. Estrella et al. demonstrated that patients undergoing surgery with microscopically positive margins have outcomes comparable to those who received more extensive resections with negative margins [90]. Surgical resection remains the treatment of choice and can often be performed as a limited pancreatic resection [58].

Cystic pancreatic neuroendocrine tumors (cystic PanNETs) are particularly challenging to diagnose based solely on radiographic imaging due to overlapping features and variable malignant potential. Given these uncertainties, active surveillance may be hazardous, and surgical resection is generally considered the preferred management strategy [66].

## 11. Conclusions

The management of pancreatic cystic lesions (PCLs) remains a major clinical challenge, driven by the need to balance early malignancy detection with the avoidance of unnecessary surgical morbidity. While the ongoing evolution of international guidelines has significantly improved risk stratification, the heterogeneity of PCLs highlights the need for more personalized diagnostic approaches.

Radiomics is emerging as a revolutionary support technology in this field, being able to transform conventional medical images into objective quantitative data, allowing for the identification of tumor heterogeneity features invisible to the human eye, thereby facilitating more precise and non-invasive diagnostics. Although there are still some technical challenges regarding the lack of methodology standardization and external validation, its potential is undeniable.

In conclusion, the future of PCL management lies in a multidisciplinary approach that integrates radiological findings, clinical and laboratory data, and established guidelines with AI-driven texture analysis. This synergy will overcome the limits of subjective evaluation, leading toward a tailored medicine specifically designed for the individual patient.

## Figures and Tables

**Table 1:** Worrisome features and High-Risk Stigmata according to 2024 Kyoto Guidelines.

Worrisome Features	High-Risk Stigmata
cyst ≥ 3 cm	MPD ≥ 10 mm
abrupt change in caliber of pancreatic duct with distal pancreatic atrophy acute pancreatitis	enhancing mural nodule ≥ 5 mm or solid component
increased serum level of CA19.9	
new onset or acute exacerbation of DM within the past year	Suspicious or positive results of cytology (if performed)
enhancing mural nodule < 5 mm thickened/enhancing cyst walls MPD ^1^ ≥ 5 mm and <10 mm lymphadenopathy cystic growth rate ≥ 2.5 mm/year	obstructive jaundice in a patient with a cystic lesion of the pancreatic head.

^1^ MPD: Main pancreatic duct.

## Data Availability

No new data were created or analyzed in this study.

## Abbreviations

The following abbreviations are used in this manuscript:

CEA	Carcinoembryonic Antigen
CECT	Contrast-Enhanced Computed Tomography
CT	Computed Tomography
DM	Diabetes Mellitus
EUS	Endoscopic Ultrasound
FNA	Fine-Needle Aspiration
HGD	High-Grade Dysplasia
HRS	High-Risk Stigmata
IPMN	Intraductal Papillary Mucinous Neoplasm
MCN	Mucinous Cystic Neoplasm
MDCT	Multidetector Computed Tomography
MPD	Main Pancreatic Duct
MRCP	Magnetic Resonance Cholangiopancreatography
MRI	Magnetic Resonance Imaging.
PanNET	Pancreatic Neuroendocrine Tumor
PCL	Pancreatic Cystic Lesion
SCN	Serous Cystic Neoplasm
SPEN	Solid Pseudopapillary Epithelial Neoplasm
T1w	T1-weighted
T2w	T2-weighted
TA	Texture Analysis
WF	Worrisome Feature
